# Evaluation of multiple biological indicators for the combined diagnosis of metastases from colorectal cancer—a retrospective study based on 1163 patients

**DOI:** 10.1186/s12957-023-03108-4

**Published:** 2023-07-28

**Authors:** Bangquan Chen, Jiajie Zhou, Yue Ma, Qiannan Sun, Jun Ren, Daorong Wang

**Affiliations:** 1 Northern Jiangsu People’s Hospital Affiliated to Yangzhou University, Yangzhou, 225001 China; 2 General Surgery Institute of Yangzhou, Yangzhou University, Yangzhou, 225001 China; 3 Yangzhou Key Laboratory of Basic and Clinical Transformation of Digestive and Metabolic Diseases, Yangzhou, China; 4 Northern Jiangsu People’s Hospital, Clinical Teaching Hospital of Medical School, Nanjing University, Yangzhou, 225001 China; 5 Medical Research Center of Northern Jiangsu People’s Hospital, Yangzhou, China; 6 Department of Gastrointestinal Surgery, Northern Jiangsu People’s Hospital, Yangzhou, 225001 Jiangsu Province China

**Keywords:** Colorectal cancer, Metastases, Biological indicators, Diagnosis

## Abstract

**Objective:**

This study aimed to investigate the efficacy of inflammatory markers (NLR, PLR) combined with tumor markers (CA50, CA199, CEA) in the diagnosis of colorectal cancer metastasis by a single-center retrospective study.

**Methods:**

A total of 1163 CRC patients who received treatments in our hospital from January 2017 to December 2021 were enrolled retrospectively. Patients were grouped according to the absence of metastasis. The separate efficacy of tumor markers, NLR and PLR, was evaluated in the diagnosis of metastasis of colorectal cancer using ROC curve analysis, and their optimal cut-off values for distant metastases from colorectal cancer were determined. The area under the ROC curve (AUC) of the tumor markers combined with NLR and PLR was calculated by binary logistic regression analysis to evaluate the diagnostic efficacy of metastasis of colorectal cancer. In addition, patients were divided into two groups of high and low levels according to the optimal cut-off values, and the effects of NLR, PLR, and tumor markers on distant metastasis of colorectal cancer were evaluated using multiple logistic regression analysis.

**Result:**

The abnormal rate of CA50, CA199, CEA, NLR, and PLR in two subgroupsIt was statistically significant (*P* < 0.05). After AUC testifying, the diagnostic efficacy of NLR and PLR was equivalent to that of tumor marker (*P* > 0.05). In assessment of liver metastasis, peritoneal metastasis, and multiple metastasis, AUC of NLR and PLR with CRC-specific tumor markers showed higher predictive efficacy than AUC without combined NLR nor PLR. The CA50, CA199, CEA, PLR, and NLR were proved independently associated with metastasis using multiple logistic regression analysis (*P* < 0.05).

**Conclusion:**

NLR and PLR are noted tumor markers of colorectal cancer, which are characterized by noninvasive, high diagnostic efficacy, easy availability, and low cost. They can be combined with traditional tumor markers to evaluate and diagnose colorectal cancer metastasis by clinicians.

**Supplementary Information:**

The online version contains supplementary material available at 10.1186/s12957-023-03108-4.

## Introduction

Colorectal cancer (CRC) is the world’s fourth most common malignant tumor. Colorectal cancer has high incidence and mortality globally, with over 1.8 million new cases and 910,000 deaths reported annually in 2020 [1]. Of those diagnosed with colorectal cancer, 20% have metastasis, and 40% have recurrence after treating primary lesions [2]. The prognosis for metastatic colorectal cancer is disappointing, with a less than 20% 5-year survival rate [3]. It is crucial to recognize the situation of metastasis in advance because it can ascertain the right time of surgery and then initiate prior chemotherapy for the patients who are inappropriate to surgery. Early recognition of the metastasis can improve the promptness and success rate of surgery.

Up to now, the pervasive methods to screen out the pre-op metastatic CRC are the following ones: CT, PET-CT, and MRI [4]. Nevertheless, taking into consideration the radiation exposure, expense, and economic burden of patients, it is of difficulty to screen out the metastasis in those ways above. So, a novel early biomarker is under urgency in clinical practice.

In recent years, increasing studies have shown that inflammation is closely related to the occurrence and progression of cancer. Inflammation is considered a hallmark feature of initiating and promoting carcinogenesis, involving every step of tumorigenesis [5, 6]. In clinical practice, conventional biomarkers reflecting systemic inflammation are circulating blood cells (e.g., neutrophils, lymphocytes and monocytes, platelets) and acute-phase proteins such as C-reactive protein (CRP). As a combination of several inflammatory cells, neutrophils-to-lymphocytes ratio (NLR) and platelets-to-lymphocytes ratio (PLR) are considered to be closely related in tumor infiltration [7], recurrence [8], metastasis [9], prognosis [10], etc. In recent years, NLR and PLR have been widely used as prognostic indicators of tumors and as predictors of early tumor diagnosis [11–14]. Nevertheless, few studies focused on the link between blood cell levels and tumor metastasis, and no study has yet verified the relationship between blood cell levels and colorectal cancers metastasis. Hereby, we conducted a retrospective study, to investigate the diagnostic efficacy of NLR and PLR on CRC metastasis.

## Material and methods

### Baseline characteristics

The clinical data of colorectal cancer patients admitted to our hospital from January 2017 to December 2021 were enrolled in our study and analyzed retrospectively.

Inclusion criteria are as follows:

1. Colorectal cancer was diagnosed by pathological examination.
2. Routine blood examination and tumor markers were performed before surgery.

Exclusion criteria are as follows:

1. Concurrent or secondary to other malignant tumors.
2. Underwent chemoradiotherapy before surgery.
3. Preoperative complications: Acute injury, acute and chronic inflammation, kidney disease, liver disease, liver disease, blood disease, and autoimmune disease.
4. Administration of anticoagulants, acetylsalicylic acid, or statins 3 months previous to surgery.

Blood parameters are the ones closest to the operative time. All methods were performed in accordance with relevant guidelines and regulations, and informed consent was obtained from all subjects and/or their legal guardians. This study was approved by the Ethics Committee of Northern Jiangsu People’s Hospital.

### Methods

When the patient was admitted, a fasting venous blood sample (2 mL) was taken into an EDTA-K2 anticoagulant tube and immediately delivered to the laboratory for whole blood cells and tumor marker testing. We recorded the parameters of the total number of leukocytes, absolute neutrophils, absolute lymphocytes, absolute monocytes, absolute hemoglobin, total platelets, and absolute values of tumor markers (CA50, CA199, CEA) [15].

The NLR values were defined by the absolute values of neutrophils-to-lymphocytes ratio. PLR values were defined by the total platelet counts to absolute lymphocytes ratio. Sensitivity, specificity, positive predictive value, negative predictive value, and area under the ROC curve (AUC) were calculated by receiver operating characteristic curve (ROC). The optimal cut-off values for NLR, PLR, and CRC tumor markers were obtained by ROC analysis [16].

Among them, the optimal cut-off values of NLR, PLR, and CRC tumor markers were determined by ROC analysis. The diagnostic value of NLR, PLR, CA50, CA199, and CEA as well as collaborative utility of them obtained by binary logistic regression was evaluated by area under the ROC curve (AUC) analysis to predict the probability. Area under the ROC (AUC) was compared using the DeLong test [17]. The optimal cut-off values for NLR, PLR, and CRC biomarkers were verified using ROC curves, and then subgroups were divided by these optimal cut-off values. Multiple logistic analysis was performed to assess the effect of clinicopathological parameters on colorectal cancer metastasis [18]. The correlation of NLR and PLR with other clinical indicators was analyzed by Spearman correlation.

### Statistical analysis

All statistical analyses were performed using the SPSS 22.0 software. Categorical data are shown by rate (%), and comparisons were performed using *χ*^2^ or Fisher test. Measurement data are shown by mean ± standard deviation, and comparisons were performed using *F* or *t*-test. *a* = 0.05 is defined as the test level.

## Result

### Demographics characteristics

A total of 1163 patients were enrolled. Six-hundred twenty-three of them were male, and 540 were female; patient’s age ranged from 28 to 99 years old, with an average age of 63.9 ± 12.8 years. Of all patients, 897 of them were diagnosed with primary tumors, 58 of them were found combined with peritoneal metastases, 18 of them were found combined with lung metastases, 160 of them were found combined with liver metastases, and 30 of them were found combined with multisite metastases. CA50, CA199, and CEA of metastasis subgroup were significantly higher than the non-metastasis subgroup (*P* < 0.001). Absolute neutrophil counts and platelet counts were found, increased and absolute lymphocyte counts were found decreased in the metastasis group, and the NLR and PLR were significantly elevated (*P* < 0.001) (Table 1).

**Table 1:** Demographics and baseline characteristics

	**Non-metastasis (*N* = 897)**	**Metastasis (*N* = 266)**	***p*-value**
**Sex**			0.657
Male	413 (46%)	127 (47.7%)	
Female	484 (54%)	139 (52.3%)	
**Age (years)**	63.7 (12.8)	64.7 (12.6)	0.269
**Erythrocyte (10**^**12**^**/L)**	4.37 (0.543)	4.25 (0.506)	< 0.001
**Hemoglobin (g/L)**	125 (23.4)	121 (19.7)	0.011
**Leukocyte (10**^**9**^**/L)**	6.12 (2.96)	6.53 (1.86)	0.007
**Neutrophil (10**^**9**^**/L)**	4.05 (1.58)	4.67 (1.78)	< 0.001
**Lymphocyte (10**^**9**^**/L)**	1.61 (0.538)	1.36 (0.558)	< 0.001
**Platelet (10**^**9**^**/L)**	219 (78.9)	245 (89.7)	< 0.001
**CA50 (U/ml)**	12.9 (32.1)	36.9 (57.8)	< 0.001
**CA199 (U/ml)**	26.8 (85.5)	137 (288)	< 0.001
**CEA (ng/ml)**	11.2 (27.5)	61.1 (166)	< 0.001
**PLR**	153 (87.3)	206 (109)	< 0.001
**NLR**	2.83 (1.73)	4.10 (2.78)	< 0.001

Number of cases, percentage or mean and standard deviation, PLR, and NLR were shown in Table 1

### NLR, PLR, and serum tumor markers predict colorectal cancer metastasis separately

Through ROC curve analysis, the best cut-off values for CA50, CA199, CEA, PLR, and NLR were 15.20 (sensitivity 44.7%, specificity 81.4%), 16.22 (sensitivity 62.0%, specificity 68.6%), 6.40 (sensitivity 65.4%, specificity 68.2%), 139.84 (sensitivity 72.6%, specificity 56.1%), and 2.74 (sensitivity 67.7%, specificity 62.9%), respectively. The AUC of CA50 for predicting colorectal cancer metastasis was 0.648 (95% *CI*: 0.608–0.687); the AUC of CA199 was 0.671 (95% *CI*: 0.632–0.711); the AUC of CEA was 0.695 (95% *CI*: 0.658–0.731); the AUC of PLR was 0.677 (95% *CI*: 0.642–0.713); and the AUC of NLR was 0.690 (95% *CI*: 0.654–0.726). Comparison of the area under ROC (AOC) using the DeLong test indicates that the inflammatory markers PLR and NLR had comparable predictive effects to serum tumor markers in predicting CRC metastasis (*P* > 0.05) (Table 2).

**Table 2:** Comparison of *P*-values for NLR, PLR, and tumor markers using the DeLong test

	AUC	95 *CI*%	*p*-value	
CA50	0.648	0.608–0.687	0.253	0.009
CA199	0.671	0.632–0.711	0.820	0.471
CEA	0.695	0.658–0.731	0.494	0.854
PLR	0.677	0.642–0.713	Ref	-
NLR	0.690	0.654–0.726	-	Ref

### The combination of NLR, PLR, and serum tumor markers predicts colorectal cancer metastasis

**Fig. 1:**
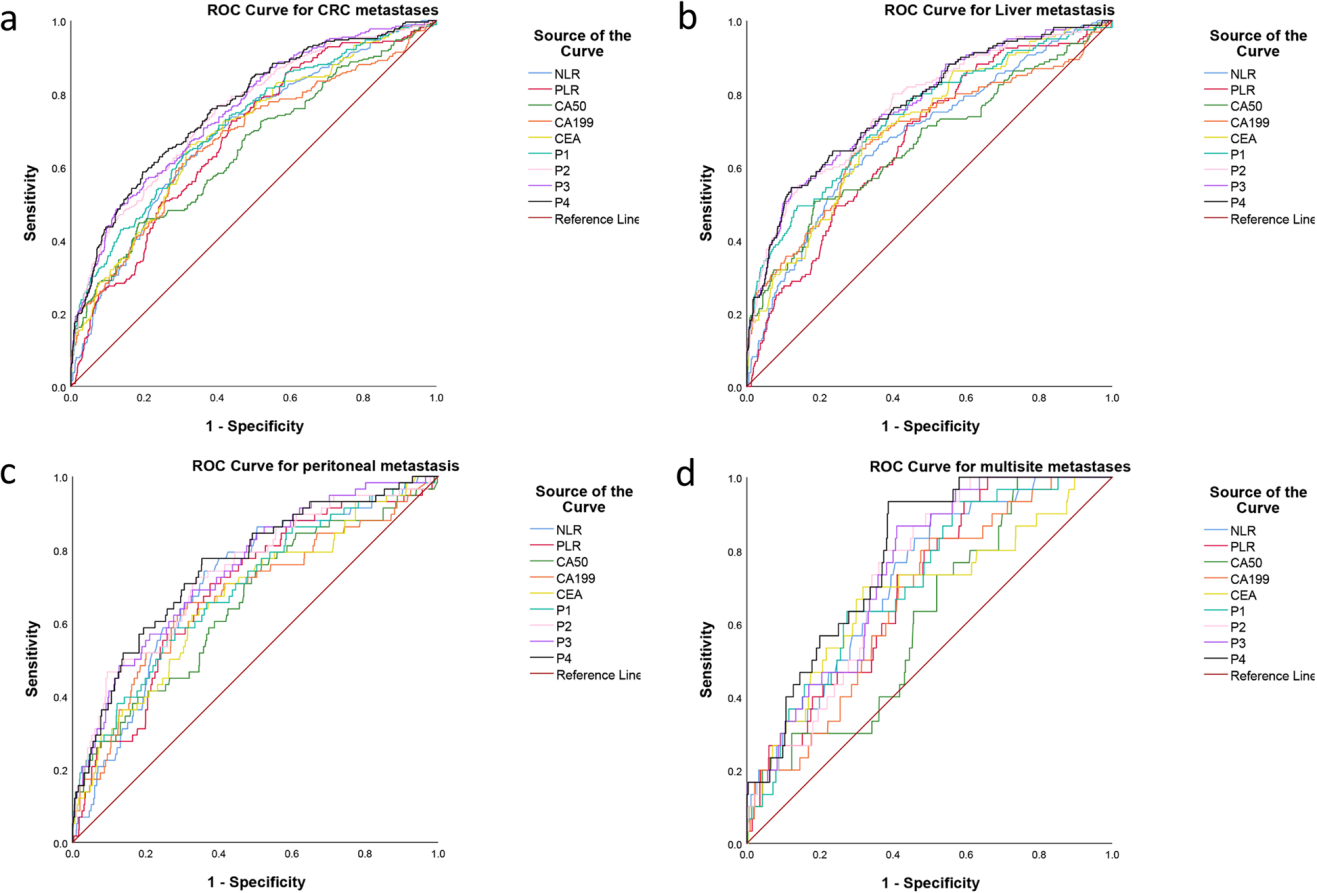
ROC analysis for predicting colorectal cancer metastasis. AUC represents the diagnostic capacity of CA50, CA199, CEA, PLR, and NLR and the predicted probability of total (**a**), liver (**b**), peritoneal (**c**), and multiple metastasis (**d**)

Predictive probability of combined CRC tumor markers (P1), CRC tumor markers combined with PLR (P2), CRC tumor markers combined with NLR (P3), and CRC tumor markers combined with PLR and NLR (P4) were calculated using binary logistic regression (Fig. 1a). AUC for P1 was 0.716 (95% *CI*: 0.680–0.752, *P* < 0.001), AUC for P2 was 0.749 (95% *CI*: 0.715–0.782, *P* < 0.001), AUC for P3 was 0.752 (95% *CI*: 0.719–0.785, *P* < 0.001), and AUC for P4 was 0.761 (95% *CI*: 0.728–0.794, *P* < 0.001). The optimal cutoff for P1 was 0.19 (sensitivity 63.5%, specificity 69.1%), the optimal cutoff for P2 was 0.17 (sensitivity 76.7.%, specificity 60.3%), the optimal cutoff for P3 was 0.21 (sensitivity was 56.8%, specificity was 79.5%), and the optimal cutoff for P4 was 0.21 (sensitivity 58.6% and specificity 80.4%). Comparison of the area under the ROC (AUC) using the DeLong test showed that combining NLR and PLR (P4) significantly improved diagnostic efficacy, except for P3 (*P* = 0.146) (Fig. 1a, Table 3).

**Table 3:** *AUC* area under the subject operating characteristics, *CI* confidence interval, *Sen* sensibility, *Spe* specificity, Youden index, *PPV* positive predictive value, *NPV* negative predictive value, *P*-values for comparisons using the DeLong test. Predictive probability of combined CRC tumor markers (P1), CRC tumor markers combined with PLR (P2), CRC tumor markers combined with NLR (P3), and CRC tumor markers combined with PLR and NLR (P4) were calculated using binary logistic regression, *Ref* reference

	AUC	95% *CI*	Cutoff	Sen	Spe	Youden index	PPV	NPV	*p*-value
CA50	0.648	0.608–0.687	15.20	0.447	0.814	0.261	0.416	0.832	< 0.001
CA199	0.671	0.632–0.711	16.22	0.620	0.686	0.306	0.369	0.859	< 0.001
CEA	0.695	0.658–0.731	6.400	0.654	0.682	0.336	0.379	0.869	< 0.001
PLR	0.677	0.642–0.713	139.8	0.726	0.561	0.287	0.329	0.873	< 0.001
NLR	0.690	0.654–0.726	2.735	0.677	0.629	0.306	0.353	0.866	< 0.001
P1	0.716	0.680–0.752	0.189	0.635	0.691	0.326	0.379	0.865	0.010
P2	0.749	0.715–0.782	0.175	0.767	0.603	0.370	0.364	0.897	0.0495
P3	0.752	0.719–0.785	0.208	0.568	0.795	0.363	0.451	0.861	0.146
P4	0.761	0.728–0.794	0.208	0.586	0.804	0.390	0.470	0.868	Ref

### Subgroup analysis of single and multiple metastases

**Fig. 2:**
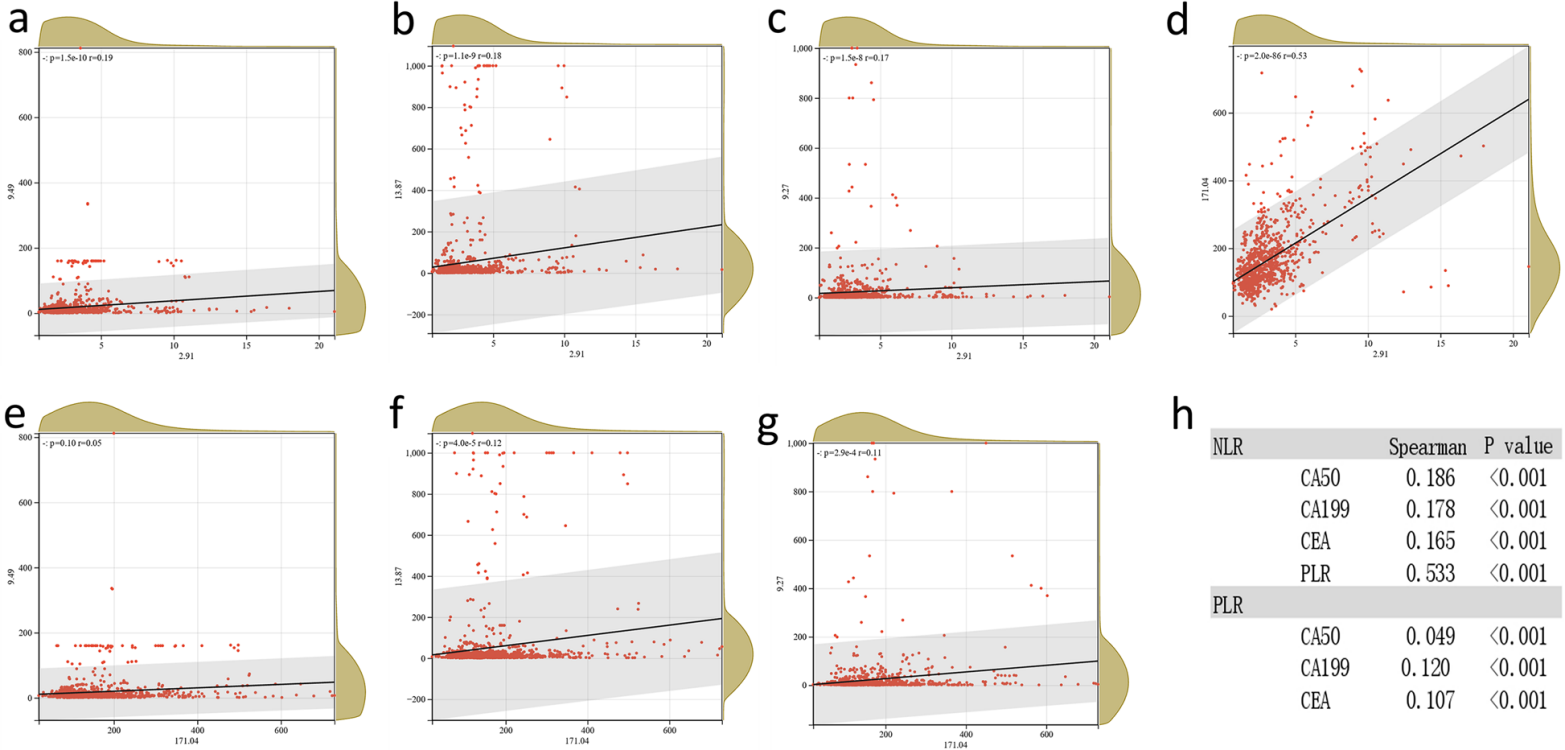
Correlation of NLR and PLR with clinicopathological features. **a** NLR-CA50. **b** NLR-CA199. **c** NLR-CEA. **d** NLR-PLR. **e** PLR-CA50. **f** PLR-CA199. **g** PLR-CEA. **h** Summary

We also stratified by metastatic sites and evaluated the diagnostic ability of PLR and NLR to detect both single-site metastasis and multisite metastasis using ROC curves. For liver metastases (*n* = 160), AUC for CA50 was 0.663 (95% *CI* 0.612–0.713), AUC for CA199 was 0.688 (95% *CI* 0.638–0.738), AUC for CEA was 0.709 (95% *CI* 0.665–0.753), AUC for PLR was 0.671 (95% *CI* 0.627–0.715), and AUC for NLR was 0.684 (95% *CI* 0.638–0.730). The diagnostic efficacy of the predicted probability (P4) of a combination of CA50, CA199, CEA, and PLR and NLR was significantly higher than the predicted probability of individual indicators (CA50, CA199, CEA, PLR, NLR, *P* < 0.05). There was no significant difference between the partial combined prediction probability (P1, P2, P3) and the AUC of P4 (*P* > 0.05), and the combination of combined PLR and NLR slightly improved the diagnostic efficacy though (Fig. 1b). For peritoneal metastasis (*n* = 58), AUC for CA50 was 0.651 (95% *CI* 0.574–0.728), AUC for CA199 was 0.672 (95% *CI* 0.593–0.751), AUC for CEA was 0.669 (95% *CI* 0.595–0.743), AUC for PLR was 0.688 (95% *CI* 0.618–0.757), and AUC for PLR was 0.706 (95% *CI* 0.641–0.772). The diagnostic efficacy of the predicted probability of CA50, CA199, CEA, and PLR and NLR was significantly higher than the single indicator, except CA199 (*P* = 0.0595) (Fig. 1c). For multiple metastases (*n* = 30), AUC for CA50 was 0.611 (95% *CI* 0.521–0.701), AUC for CA199 was 0.665 (95% *CI* 0.579–0.750), AUC for CEA was 0.679 (95% *CI* 0.573–0.785), AUC for PLR was 0.700 (95% *CI* 0.623–0.778), and AUC for NLR was 0.724 (95% *CI* 0.645–0.803). Similar to peritoneal metastasis, the diagnostic efficacy of the predicted probability was significantly higher than that for individual indicators, except for CEA (*P* = 0.0909) and PLR (*P* = 0.0581) (Fig. 1d, Supplementary Table 1). Due to the small sample size, we did not perform the analysis of lung metastasis (*n* = 18). In addition, the correlation analysis of NLR and PLR with other parameters showed that NLR and PLR were positively correlated with CA50, CA199, and CAE in CRC patients (*r* = 0.186, 0.178, 0.10, 0.165 and 0.049, 0.120, 0.107, respectively, *P* < 0.001), with the highest correlation with PLR (*r* = 0.533, *P* < 0.001) (Fig. 2).

### Univariate and multivariate logistic regression analysis of distant metastases in colorectal cancer

**Fig. 3:**
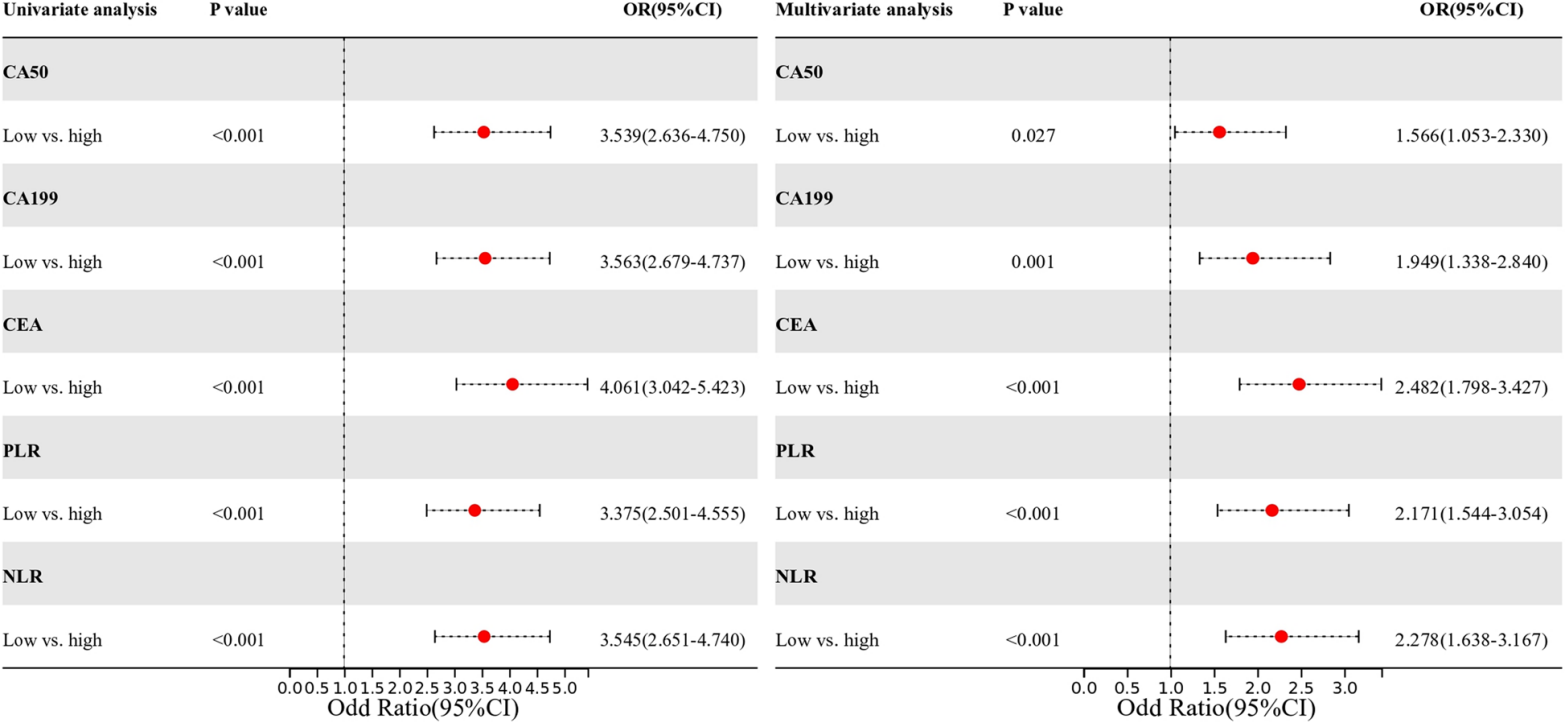
Univariate and multivariate logistic regression analysis of colorectal cancer metastasis in forest plots

Grouped by parameter optimal cut-off value. Multivariate logistic regression analysis revealed the following: higher NLR (*OR*: 2.278, 95% *CI*: 1.638–3.167, *P* < 0.001), PLR (*OR*: 2.171, 95% *CI*: 1.544–3.054, *P* < 0.001), CA50 (*OR*: 1.566, 95% *CI*: 1.053–2.330, *P* = 0.027), CA199 (*OR*: 1.949, 95% *CI*: 1.338–2.840, *P* = 0.001), and CEA (*OR*: 2.482, 95% *CI*: 1.798–3.427, *P* < 0.001) were independent risk factors for CRC metastasis. Results were demonstrated in forest plots (Fig. 3).

## Discussion

Increasing evidence suggests that inflammatory responses play an important role in tumor progression. Epidemiological studies have found that more than 15% of human cancers worldwide are caused by chronic inflammation [19]. Neutrophils, lymphocytes, platelets, and monocytes are involved in tumor development and progression as important components of the microenvironment [20]. Neutrophils have been reported to modulate the tumor microenvironment and enhance angiogenesis, proliferation of tumor cells, metastasis, and their evasion of immune responses [21]. Lymphocytes play an important role in antitumor immunity by mediating tumor cell-specific apoptosis, thereby inhibiting the progression of tumor cells [22]. Platelets, acting as dynamic reservoirs of various factors, stimulate tumor cell proliferation and enhance angiogenesis by secreting large amounts of cytokines and growth factors. Tumors protect the inner environment by secreted factors that retain platelets to positively influence their survival. Moreover, tumor cells can induce platelet aggregation, the so-called tumor cells, which enables tumor cells to evade immune surveillance and protect tumor cells from cytolysis [23, 24].

Given that tumor patients have trends to have neutrophil and thrombocytosis and lymphopenia. As a combination of these inflammatory cells, NLR and PLR are relatively new inflammatory indicators, which can reflect the anti-tumor immune suppression and systemic inflammatory response, and could be regarded as the prognostic marker of tumor and the predictive index of early diagnosis of cancer. A meta-analysis of the prognostic role of NLR in breast cancer suggested that high NLR was associated with adverse OS and DFS in breast cancer patients and had a greater impact on disease-specific outcomes in ER- and HER2-negative disease [25]. Meanwhile, a meta-analysis of 100 studies containing 40,559 patients yielded similar conclusions that high NLR is a poor prognostic indicator of multiple malignancies and is associated with adverse OS in many solid tumors [26]. Moreover, many investigators have shown that PLR is closely associated with the risk of poor prognosis in various malignant tumors, including liver cancer [27], gastric cancer [28], colorectal cancer [29], nasopharyngeal carcinoma [30], lung cancer [31], and penile cancer [32].

Most of previous studies on the one hand focused on a point that high NLR and PLR could be regarded as useful predictors of long-term survival in cancer patients; on the other hand, they had explored their practical functions in the evaluation of lymph node metastasis [33–36]. Nevertheless, the roles of high NLR and PLR in assessing metastasis have not received enough attention yet. To date, it has been shown that high NLR is an essential risk factor for distant metastasis in oral squamous-cell carcinoma (*HR* = 3.122; 95% *CI*: 1.744–5.589, *P* < 0.001) [37]. In pancreatic ductal adenocarcinoma, it was found in the study of Tao et al. that NLR was a valid predictor of the onset of metastasis [38]. Moreover, in a retrospective study containing 1667 gastric cancer patients, it was also shown that NLR and PLR have good predictive efficacy in predicting metastasis [9]. In CRC, although it was suggested in few studies that high NLR was an independent risk factor for bone and liver metastasis in CRC [39, 40], but previous studies were limited to some degree, they only explored the predictive value of NLR and PLR for liver and bone metastasis in colorectal cancer. Our study explored the predictive role of this biological indicator in many aspects and compared it with traditional tumor markers in terms of diagnostic effect.

In our study, the clinical data of colorectal cancer patients admitted in recent years were analyzed retrospectively, and the subgroups criteria were determined by the occurrence of metastasis. It was indicated that NLR and PLR and tumor markers (CEA, CA199, and CA50) in the metastasis group were significantly higher than those in the non-metastasis group (*P* < 0.05), consistent to the previous studies [41, 42], indicating that preoperative NLR, PLR, and tumor indicators may be accurate diagnostic indicators for CRC metastasis. We performed ROC analysis; the optimal cut-off values for NLR, PLR, and tumor markers (CEA, CA199, and CA50) in predicting metastasis in CRC patients were 2.735, 6.400, 16.22, and 15.20, respectively; and the AUC was 0.690, 0.677, 0.695, 0.671, and 0.648, respectively. The NLR and PLR were comparable to the tumor markers CEA, CA199, and CA50 in terms of diagnostic efficacy (*p* > 0.05), indicating that NLR and PLR, like tumor markers, had certain value in predicting colorectal cancer metastasis. The collaborative utility of tumor markers (CEA, CA199, CA50), NLR and PLR, was testified by this study, demonstrating a higher diagnostic efficacy. This study sets the optimal cut-off value of NLR, PLR, and tumor makers derived from the ROC analysis as the subgroup criteria. NLR and PLR are proved to be independent predictors of CRC metastasis using univariate and multivariate logistic regression. It is clear that NLR and PLR can be used as early biomarker in patients with distant metastasis of CRC.

## Conclusion

NLR, PLR, and tumor markers (CA50, CA199, CEA) have likewise and highly efficient diagnostic efficacy in predicting colorectal metastasis. The collaborative action of NLR and PLR with tumor markers improves the specificity and sensitivity in predicting colorectal metastasis and can provide a valid reference tool for clinicians in the evaluation and diagnosis of colorectal cancer metastasis.

## Supplementary Information

**Additional file 1.**

## Data Availability

The data presented in this study are available on reasonable request.
