# Supplementary material for: Evaluation of multiple biological indicators for the combined diagnosis of metastases from colorectal cancer—a retrospective study based on 1163 patients
Source: World J Surg Oncol. 2023 Jul 28;21:229. doi: 10.1186/s12957-023-03108-4 (PMC10375667; doi:10.1186/s12957-023-03108-4)
Supplement: Supplementary file 1 — Additional file 1. [file 12957_2023_3108_MOESM1_ESM.docx]

| AUC | 95%CI | Cut-off | Sen | Spe | Youden index | PPV | NPV | P value |
| --- | --- | --- | --- | --- | --- | --- | --- | --- |
| CA50 0.663 | 0.612-0.713 | 15.2 | 0.506 | 0.814 | 0.320 | 0.327 | 0.902 | <0.001 |
| CA199 0.688 | 0.638-0.738 | 17.44 | 0.631 | 0.712 | 0.343 | 0.281 | 0.915 | 0.0033 |
| CEA 0.709 | 0.665-0.753 | 6.51 | 0.669 | 0.685 | 0.354 | 0.274 | 0.921 | 0.0099 |
| PLR 0.684 | 0.638-0.730 | 139.84 | 0.719 | 0.561 | 0.280 | 0.226 | 0.918 | <0.001 |
| NLR 0.671 | 0.627-0.715 | 3.18 | 0.594 | 0.718 | 0.312 | 0.273 | 0.908 | <0.001 |
| P1 0.737 | 0.692-0.782 | 0.11 | 0.744 | 0.612 | 0.356 | 0.255 | 0.931 | 0.1439 |
| P2 0.766 | 0.725-0.808 | 0.15 | 0.55 | 0.861 | 0.411 | 0.413 | 0.914 | 0.8809 |
| P3 0.767 | 0.726-0.808 | 0.152 | 0.544 | 0.877 | 0.421 | 0.442 | 0.915 | 0.3507 |
| P4 0.768 | 0.727-0.810 | 0.15 | 0.544 | 0.878 | 0.422 | 0.444 | 0.915 | Ref |

Liver metastasis

| AUC | 95%CI | Cut-off | Sen | Spe | Youden index | PPV | NPV | P value |
| --- | --- | --- | --- | --- | --- | --- | --- | --- |
| CA50 0.651 | 0.574-0.728 | 7.05 | 0.759 | 0.483 | 0.242 | 0.087 | 0.969 | 0.0139 |
| CA199 0.672 | 0.593-0.751 | 16.22 | 0.655 | 0.686 | 0.341 | 0.119 | 0.969 | 0.0595 |
| CEA 0.669 | 0.595-0.743 | 6.225 | 0.621 | 0.673 | 0.294 | 0.109 | 0.965 | 0.0346 |
| PLR 0.706 | 0.641-0.772 | 151.45 | 0.707 | 0.629 | 0.336 | 0.108 | 0.971 | 0.0184 |
| NLR 0.688 | 0.618-0.757 | 2.81 | 0.741 | 0.64 | 0.381 | 0.117 | 0.975 | 0.0481 |
| P1 0.694 | 0.622-0.767 | 0.05 | 0.586 | 0.72 | 0.306 | 0.119 | 0.964 | 0.0854 |
| P2 0.737 | 0.668-0.807 | 0.05 | 0.724 | 0.66 | 0.384 | 0.121 | 0.973 | 0.2768 |
| P3 0.745 | 0.681-0.810 | 0.05 | 0.69 | 0.672 | 0.362 | 0.12 | 0.971 | 0.3291 |
| P4 0.754 | 0.688-0.820 | 0.05 | 0.776 | 0.647 | 0.423 | 0.123 | 0.978 | Ref |

Peritoneal metastasis

| AUC | 95%CI | Cut-off | Sen | Spe | Youden index | PPV | NPV | P value |
| --- | --- | --- | --- | --- | --- | --- | --- | --- |
| CA50 0.611 | 0.521-0.701 | 4.97 | 1 | 0.260 | 0.260 | 0.043 | 1 | <0.001 |
| CA199 0.665 | 0.579-0.750 | 9.90 | 0.833 | 0.501 | 0.334 | 0.053 | 0.989 | 0.0128 |
| CEA 0.679 | 0.573-0.785 | 6.40 | 0.700 | 0.682 | 0.382 | 0.069 | 0.986 | 0.0909 |
| PLR 0.724 | 0.645-0.803 | 109.92 | 1 | 0.341 | 0.341 | 0.048 | 1 | 0.0581 |
| NLR 0.7 | 0.623-0.778 | 2.36 | 0.508 | 0.900 | 0.408 | 0.056 | 0.993 | 0.0047 |
| P1 0.714 | 0.631-0.797 | 0.027 | 0.933 | 0.431 | 0.364 | 0.052 | 0.995 | 0.1826 |
| P2 0.733 | 0.666-0.799 | 0.027 | 0.800 | 0.625 | 0.425 | 0.067 | 0.989 | 0.1742 |
| P3 0.746 | 0.677-0.816 | 0.024 | 0.867 | 0.590 | 0.457 | 0.066 | 0.992 | 0.0500 |
| P4 0.784 | 0.722-0.846 | 0.024 | 0.933 | 0.613 | 0.546 | 0.075 | 0.996 | Ref |

Multiple metastasis
